# Muscle mass rather than muscle strength or physical performance is associated with metabolic syndrome in community-dwelling older Chinese adults

**DOI:** 10.1186/s12877-021-02143-8

**Published:** 2021-03-19

**Authors:** Peiyu Song, Peipei Han, Yinjiao Zhao, Yuanyuan Zhang, Liyan Wang, Zhuoying Tao, Zhengxing Jiang, Shijing Shen, Yunxiao Wu, Jiajie Wu, Xiaoyu Chen, Xing Yu, Yong Zhao, Qi Guo

**Affiliations:** 1 Department of Rehabilitation Medicine, Shanghai University of Medicine and Health Sciences Affiliated Zhoupu Hospital, 1500 Zhouyuan Road, Pudong New District, Shanghai, 201318 China; 2 Department of Rehabilitation Medicine, Tianjin Medical University, Tianjin, China; 3 Tianjin Key Laboratory of Metabolic Cardiovascular Diseases, Key Laboratory of Immune Microenvironment and Disease-Ministry of Education, Department of Physiology and Pathophysiology, Tianjin Medical University, Tianjin, China

**Keywords:** Metabolic syndrome, Muscle mass, Muscle strength, Physical performance

## Abstract

**Objective:**

The purpose of this study was to examine whether muscle mass, muscle strength, and physical performance were associated with metabolic syndrome (MetS) in community-dwelling older Chinese adults.

**Methods:**

The study comprised of 1413 community-dwelling Chinese participants (577 men; mean ± standard deviation age: 71.3 ± 5.9) recruited from Tianjin and Shanghai, China who were invited to participate in a comprehensive geriatric assessment. The International Diabetes Federation metabolic syndrome guidelines were used to define MetS, including high waist circumference, elevated blood pressure, elevated fasting blood glucose, elevated triglycerides, and reduced HDL cholesterol. Muscle mass was measured by appendicular skeletal muscle mass/weight (ASM/weight), and ASM was measured by BIA. Muscle strength was measured using grip strength. Physical performance was represented by walking speed and the time up and go test (TUGT).

**Results:**

The overall prevalence of MetS was 46.8% (34.1% in males and 55.5% in females). In the final logistic regression model, there was a significant, graded inverse association between muscle mass and MetS (p for trend = 0.014). Muscle strength and physical performance, including walking speed and TUGT, were not associated with overall MetS. In the components of MetS, muscle mass and grip strength were significantly inversely associated with high waist circumference and elevated blood pressure (*p* < 0.05), while physical performance was not associated with components of MetS.

**Conclusions:**

Compared with muscle strength and muscle function, muscle mass was inversely associated with MetS in a community-dwelling elderly Chinese population. Among muscle mass、muscle strength and physical performance, muscle mass appears to have the strongest association with MetS in the elderly.

**Supplementary Information:**

The online version contains supplementary material available at 10.1186/s12877-021-02143-8.

## Introduction

Metabolic syndrome (MetS) is a cluster of cardiovascular risk factors, including central obesity, elevated blood pressure, elevated fasting blood glucose, and dyslipidemia. These factors have been proven to be significantly related to cardiovascular disease and all-cause mortality. Epidemiological evidence indicates that the incidence of MetS increases dramatically with age [1], and the risk of MetS in elderly people over 60 years old is 2–3 times higher than that of adults [2, 3]. In China, aging is a severe challenge as the Chinese population over 60 years old reached 230 million in 2016. This number continues to increase, and is expected to be approximately 418 million by 2035 [4].

One of the most dramatic changes in the elderly aging process is the decline of muscle mass and function, As the main component of sarcopenia, it has been studied more and more [5]. Skeletal muscle is the main part of carbohydrate and fatty acid metabolism, and its endocrine function plays a key role in reversing metabolic disorder [6]. Muscle activity also complements skeletal muscle’s adaptation in a multitude of signal pathways to maintain metabolic balance by enhancing glucose and lipid metabolism and endocrine activity [7]. Thus, skeletal muscle may play an important role in the development of MetS. Some studies have shown that low muscle mass is a risk factor for MetS [8], while others have found no such association [9]. Identifying the association between muscle characteristics and MetS may provide a new pathway for the treatment of MetS. Moreover, to the best of our knowledge, no studies have been performed on the relationship between muscle mass and MetS in the elderly Chinese population. Muscle function includes both muscle strength and physical performance [10]. Although the relationship between muscle strength and physical performance and MetS has been widely studied, no conclusive consensus on these associations has been reached [11–14]. It is worth noting that muscle strength has been proven to be an important biomarker of cardiovascular disease, cardiovascular death, and all-cause death [15].

Therefore, the purpose of this study was to determine the association between muscle mass and function and MetS among community-dwelling elderly Chinese individuals, which account for about 70% of the elderly in China. In addition, the association between muscle mass and function and MetS was compared.

## Methods

### Study participants

The present study is a cross-sectional analysis, using data from Adult Physical Fitness and Health Cohort Study (APFHCS) [ChiCTR1900024880], APFHCS is a large prospective dynamic cohort study, mainly investigated the association between physical fitness and health status in a general adult population living in Tianjin and Shanghai, China. Participants were recruited for taking annual comprehensive health examinations and completed detailed questionnaires regarding their lifestyle and disease history. The data of this study were taken from August 2018 to July 2019. The survey is for comprehensively understanding the health testing indicators and evaluation systems of the elderly (age 60 and above). Prior to the survey, we explained the consent process, study procedures and purpose to the participant. The inclusion criteria were willingness to participate and cooperate with relevant inspections in the study. Exclusion criteria of this study were as follows: (1) age < 60 years; (2) unable to communicate with interviewers or to grant the informed consent; (3) history of condition, such as hand injury, which influences HS level; and (4) inability to stand upright for measurements of body composition, weight and height; (5) unable to perform the time up and go test or the 4-m walking test; (6) no blood samples were collected.

**Fig. 1:**
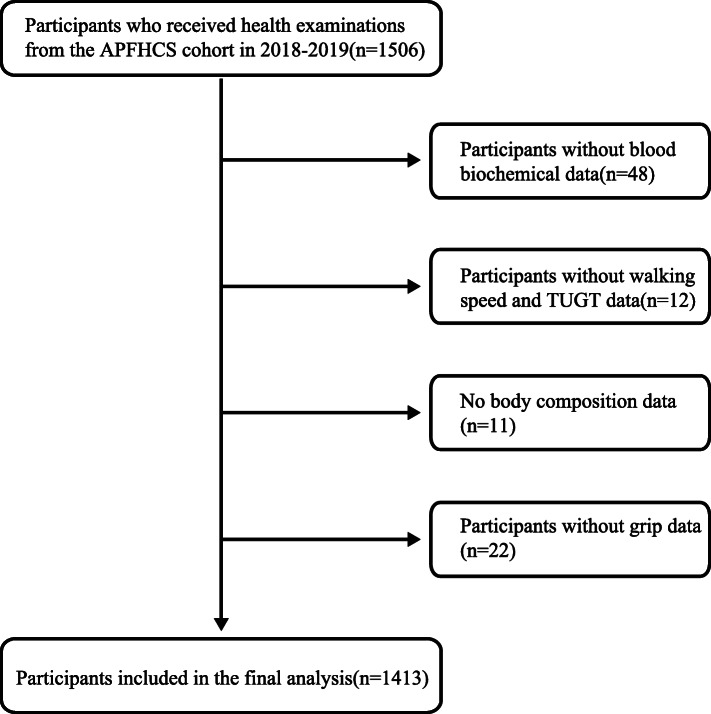
Flowchart of the study population selection process

A total of 1506 people whom resides in Hangu District, Tianjin and Chongming District Shanghai, took part in the national free physical examination program as part of a comprehensive geriatric assessment. A flow chart detailing the derivation of sample is presented in Fig. 1. Due to missing values or criteria it was not possible to analyze 93 participants meaning 1413 are available for analyses. Our study was approved by the Ethics Committee of Tianjin Medical University and Shanghai University of Medicine and Health Sciences and its participants have provided informed consent prior to participation.

### Baseline variable

All the participants were invited to a face-to-face interview to answer a standardized questionnaire after they completed their medical examination. The questionnaire included questions about age, sex, occupation, educational level, marital status (with spouse, widowed, unmarried, divorced), family income (< 1000 yuan, 1000–3000 yuan, 3000–5000 yuan,> 5000 yuan), smoking habits (current smoker or not), drinking habits (drinking alcohol once a week, drinking in the past, and never drinking were all considered as no drinking). Physical activity was assessed using the short form of the International Physical Activity Questionnaire (IPAQ) [16]. Nutritional status assessment using the Mini Nutritional Assessment-short form (MNA-SF) [17]. We also reviewed whether participants had chronic medical conditions, such as type 2 diabetes mellitus (T2DM), hypertension, hyperlipidemia, cardiovascular disease (CVD), stroke, kidney disease and cancer.

### Anthropometric measurements

Bioimpedance analysis (In-Body720; Biospace Co., Ltd., Seoul, Korea) was used for estimating appendicular skeletal mass (ASM). The skeletal muscle mass index (SMI) was calculated as follows: SMI (%) = total ASM (kg)/body weight (kg) × 100 [18], which was modified from the study of Janssen et al. [19].

### Analysis of blood samples and blood pressure

A blood sample was obtained from the antecubital vein from patients who fasted overnight for at least 10 h. Blood sample analysis and blood pressure collection methods have been explained in our previous studies [20].

### Performance-based assessment

Performance-based assessment consisted of several physical tests (4-m walking velocity test and the Timed Up and Go Test (TUGT)) and grip strength. Detailed test methods have been described in our previous studies [21].

### Definition of MetS

According to the International Diabetes Federation (IDF), people with MetS are defined by having central obesity (WC ≥ 90 cm in men and ≥ 80 cm in women) along with two or more of the following abnormalities: (1) elevated triglycerides (≥150 mg/dL); (2) reduced HDL cholesterol (< 40 mg/dL in men and < 50 mg/dL in women); (3) elevated blood pressure (≥130/85 mmHg or known treatment for hypertension); (4) elevated FPG (≥100 mg/dL, or known treatment for diabetes) [22].

### Definition of sarcopenia

Sarcopenia was defined according to the AWGS criteria [23], in which a person who has low muscle mass, low muscle strength, and/or low physical performance was identified as having sarcopenia. Low muscle mass was classified as a relative skeletal muscle mass index (ASM/height^2^) less than 7.0 kg/m^2^ and 5.7 kg/m^2^ in men and women, respectively; low muscle strength was defined as a grip strength < 26 kg or < 18 kg for males and females, respectively; low physical performance was defined as a walking speed < 0.8 m/s for both males and females.

### Statistical analysis

All continuous variables were presented as means±SD; classification variables were reported as percentages. Differences in the characteristics according to metabolic syndrome status were analyzed using t-tests, chi-square test, and Kruskale Wallis rank tests. Participants were divided into four groups by quadrisection based on the results of grip strength/weight, ASM/weight, TUGT, and WS [24, 25]. Logistic regression analysis was used to determine odds ratios (ORs) and 95% CIs and to assess whether participants with MetS were independently associated with muscle mass、muscle function and sarcopenia when compared with those without MetS. A linear trend across the quartiles of grip strength/weight, ASM/weight, TUGT, and WS were tested by using the median value of each category as an ordinal variable. Linear regression was used for calculating p for trend in the logistic binary models [26]. Several confounding factors were adjusted: age, sex, educational level, family income, smoking status, occupation, physical activity level [24]. Differences were defined as significant when *p* < 0.05. All statistical analyses were performed with the SPSS V22.0 software.

## Results

### Baseline characteristics

General characteristics of 1413 participants (577 men; mean age: 71.3 ± 5.9) are given in Table 1. The overall prevalence of MetS was 46.8% (34.1% in males and 55.5% in females). Subjects with MetS had a higher percentage of high waist circumference, high blood pressure, elevated blood glucose, and dyslipidemia than subjects without MetS (all *p* < 0.01). Moreover, subjects with MetS had significantly higher weight, BMI and incidence rate of CVD and stroke than those without MetS (all p < 0.01). However, subjects with MetS had significantly lower grip strength, SMI, and walking speed than subjects without MetS (all p < 0.01).

**Table 1:** General Characteristics of participants with and without MetS

Variable	MetS	Not MetS	P
N	662	752	
Age(y)	71.4 ± 6.0	71.1 ± 6.0	0.365
Sex			< 0.001
Male(%)	29.8	50.5	
Female(%)	70.2	49.5	
Weight(kg)	64.8 ± 10.8	59.2 ± 11.0	< 0.001
BMI(kg/m^2^)	25.6 ± 3.1	22.8 ± 3.3	< 0.001
Illiteracy (%)	20.1	17.2	0.152
Farming (%)	59.8	54.5	0.047
Smoking (%)	12.1	22.9	< 0.001
Drinking (%)	22.0	31.7	< 0.001
Grip(kg)	23.7 ± 8.8	21.6	< 0.001
Grip/weight (%)	33.3 ± 12.0	40.3 ± 13.7	< 0.001
ASM(kg)	17.9 ± 4.5	17.6 ± 4.4	0.184
SMI(%)	27.0 ± 4.2	30.1 ± 5.1	< 0.001
IPAQ(Met/wk)	5654 ± 6622	6177 ± 6665	0.053
TUGT(s)	10.6 ± 4.0	10.2 ± 7.1	0.616
WS(m/s)	1.04 ± 0.24	1.09 ± 0.24	< 0.001
MNA	11.2 ± 1.3	11.2 ± 1.2	0.639
MetS components (%)			
High WC	100	48.5	< 0.001
Elevated blood pressure	90.2	57	< 0.001
Elevated fasting glucose	67.9	28.2	< 0.001
Elevated TG	61.6	17.6	< 0.001
Low HDL	36.6	6.9	< 0.001
Chronic conditions (%)			
CVD	34.5	26.9	0.002
Stroke	26	18.4	0.001
Cancer	2.1	2.7	0.507
Kidney disease	8.5	8.0	0.736

Notes: BMI, body mass index; ASM, appendicular skeletal mass; SMI, skeletal muscle mass index; TUGT, timed up and go test; WS, 4 m walking speed; MNA, Mini-Nutritional Assessment; IPAQ, International Physical Activity Questionnaire; WC, waist circumference; MetS, metabolic syndrome. Data are presented as mean ± SD or n (%)

The association between muscle mass and muscle function and MetS is displayed in Table 2. The results showed that the elderly with lower grip strength, slower walking speed and lower SMI were more likely to suffer from MetS in the unadjusted population. In the final logistic models, subjects in the lower quartile for SMI had a significantly higher risk for MetS compared with those in the fourth quartile and were as follows: 0.67 (0.45–1.00); 0.27 (0.15–0.47); and 0.04 (0.01–0.14) (p for trend< 0.05).

**Table 2:** Logistic regression analyses of the association of grip, SMI, TUGT, and walking speed quartiles with MetS

	Q1	Q2	Q3	Q4	P for trend
Grip/weight(%)					
N	353	353	354	353	
Crude	Ref	0.88 (0.65–1.19)	0.55 (0.41–0.75)	0.23 (0.17–0.32)	0.012
Adjusted model	Ref	0.98 (0.63–1.52)	0.40 (0.25–0.64)	0.37 (0.21–0.65)	0.106
SMI (%)					
N	350	354	354	385	
Crude	Ref	0.68 (0.50–0.93)	0.48 (0.36–0.66)	0.12 (0.09–0.18)	0.009
Adjusted model	Ref	0.67 (0.45–1.00)	0.27 (0.15–0.47)	0.04 (0.01–0.14)	0.014
WS (m/s)					
N	352	354	354	353	
Crude	Ref	0.80(0.59–1.07)	0.73 (0.54–0.98)	0.59 (0.43–0.79)	0.004
Adjusted model	Ref	0.71(0.44–1.15)	0.75 (0.45–1.24)	0.71 (0.42–1.21)	0.181
TUGT (s)					
N	353	354	354	352	
Crude	Ref	1.60 (1.18–2.16)	1.34 (0.99–1.81)	1.66 (1.23–2.25)	0.270
Adjusted model	Ref	1.42 (0.85–2.37)	1.13 (0.67–1.89)	1.09 (0.64–1.87)	0.247

Data are presented as adjusted OR, with 95% CI in parentheses, unless otherwise stated

Adjusted model is adjusted with age, sex, smoking status, drinking, occupation, MNA, educational level, family income and physical activity level, CHD, stroke

*Notes*: *SMI* skeletal muscle mass index, *TUGT* timed up and go test, *WS* 4 m walking speed, *MNA* Mini-Nutritional Assessment, *MetS* metabolic syndrome, *Q* quartiles

The association between muscle mass and muscle function and MetS is displayed in Table 3. After adjustment, there was no significant association between sarcopenia and metabolic syndrome and its components.

**Table 3:** Logistic regression analyses of the association of sarcopenia with MetS and its components

Variable	Odds ratio(95%CI)			
	Crude	P	Adjusted model	P
Sarcopenia				
Metabolic syndrome	0.48(0.34–0.68)	< 0.001	1.08(0.70–1.66)	0.726
High waist circumference	0.24(0.17–0.33)	< 0.001	1.20(0.58–2.48)	0.630
Elevated blood pressure	0.74(0.52–1.05)	0.094	1.08(0.64–1.84)	0.767
Elevated fasting glucose	1.04(0.75–1.45)	0.796	1.39(0.87–2.23)	0.170
Elevated TG	0.76(0.54–1.08)	0.126	1.07(0.64–1.77)	0.805
Low HDL	1.13(0.76–1.67)	0.556	1,67(0.95–2.94)	0.077

Data are presented as adjusted OR, with 95% CI in parentheses, unless otherwise stated

Adjusted model is adjusted with age, sex, BMI, smoking status, drinking, occupation, MNA, educational level, family income and physical activity level, CHD, stroke

*Notes*: *MNA* Mini-Nutritional Assessment, *MetS* metabolic syndrome

The relationship between SMI, grip strength, walking speed, TUGT and the components of MetS is shown in Table 4. In the adjusted logistic regression model, both lower muscle mass and lower grip strength were significantly associated with an increased risk of high waist circumference and elevated blood pressure (*p* < 0.05), but not with other components. Walking speed and TUGT were not associated with MetS components.

**Table 4:** Logistic regression analyses of the association of grip, SMI and walking speed with MetS components

Variable	Odds ratio(95%CI)				
	High waist circumference	Elevated blood pressure	Elevated fasting glucose	Elevated TG	Low HDL
Grip/weight (%)					
Crude	0.93(0.92–0.94) **	0.98(0.97–0.99) **	0.99(0.98–0.99) **	1.00(0.99–1.00)	0.97(0.96–0.98) **
Adjusted model	0.95(0.94–0.97) **	0.98(0.96–0.99) **	1.00(0.98–1.01)	1.00(0.99–1.02)	1.00(0.98–1.02)
SMI (%)					
Crude	0.73(0.70–0.76) **	0.95(0.93–0.98) **	0.97(0.95–0.99) **	0.98(0.96–1.00)	0.88(0.85–0.91) **
Adjusted model	0.56(0.49–0.63) **	0.99(0.82–0.94) **	1.01(0.96–1.06)	0.97(0.92–1.03)	0.97(0.92–1.04)
WS (m/s)					
Crude	0.51(0.31–0.85) **	0.43(0.26–0.71**	0.58(0.37–0.90) *	0.96(0.61–1.50)	0.51(0.30–0.87) *
Adjusted model	0.84(0.35–2.00)	0.83(0.37–1.88)	0.62(0.31–1.24)	1.33(0.63–2.81)	0.62(0.26–1.49)
TUGT (s)					
Crude	1.04(1.00–1.08) *	1.01(0.98–1.04)	1.01(0.99–1.03)	0.98(0.95–1.01)	1.01(0.99–1.03)
Adjusted model	1.05(1.00–1.11)	0.98(0.96–1.00)	1.00(0.98–1.02)	0.98(0.94–1.02)	1,00(0.97–1.02)

Data are presented as adjusted OR, with 95% CI in parentheses, unless otherwise stated, *p < 0.05, **p < 0.01

Adjusted model is adjusted with age, sex, smoking status, drinking, occupation, MNA, educational level, family income and physical activity level, CHD, stroke

Notes: *SMI* skeletal muscle mass index, *TUGT* timed up and go test, *WS* 4 m walking speed, *MNA* Mini-Nutritional Assessment, *MetS* metabolic syndrome

## Discussion

The purpose of this cross-sectional study was to examine the relationship between muscle mass and function and MetS in a community-dwelling elderly Chinese population. A significant, graded inverse association between muscle mass and the risk of MetS was observed. However, although muscle strength was inversely associated with high waist circumference and high blood pressure, it was not grade inversely associated with overall MetS, and there was no association between physical performance and MetS or its components.

In previous studies, the relationship between muscle mass and MetS has not been fully established. Several studies have shown that low muscle mass is a risk factor for MetS [8, 27]; however, there are contradictions within these results. A Korean cross-sectional study of subjects over 70 years old showed that low relative muscle mass is not an independent risk factor for MetS as the association fell away after combining with central obesity [9]. Similarly, another study showed that low muscle mass was a risk factor for MetS in the non-obese elderly but not in the obese elderly [28]. Our results are consistent with these previous studies. Low muscle mass was a risk factor for MetS in non-obese community-dwelling elderly Chinese people (p for trend = 0.009), while this risk factor was not significant in obese people, see supplementary Table 2. In contradiction, studies have shown that the obese group with normal muscle mass has the highest risk of MetS among four groups stratified by obesity and low muscle mass [29]. Two reasons for the discrepancies in results from these studies may exist: first, in the overweight and obese elderly, there may be different cut-off point values for low muscle mass; second, the methods to define muscle mass indices may cause inconsistency. At present, the main definition of muscle mass is ASM adjusted by the weight or square of height. In our study, we used weight adjusted ASM as it has been shown to be more closely related to MetS and its parameters than ASM/height^2^ [30], and a stronger association between weight adjusted ASM and MetS has been reported [8, 27]. Adjusting for height does not take body fat into account, while weight includes both muscle and fat mass. Thus, it is not clear whether low muscle mass defined by ASM/weight represents relatively low muscle mass, relatively rich fat mass, or both. More precise adjustment may help to more clearly elucidate the relationship between muscle mass and MetS rather than increasing the diagnostic sensitivity. The ratio of muscle mass to visceral fat has been shown to be an important predictor of MetS [31]. Taken altogether, the relationship between muscle mass and MetS needs further study in the future.

As surrogate of muscle strength, grip strength is a proven risk stratification method for all-cause death, cardiovascular death, and cardiovascular disease [32]. Although the relationship between muscle strength and MetS has been proven by many studies, the conclusion has not reached a consensus. A Taiwanese study showed that there was no association between absolute grip strength and MetS [12], but there was a association between grip strength and MetS after weight adjustment [33]. Recently, the Foundation for the National Institutes of Health sarcopenia project has suggested that grip strength corrected by body mass index is a good marker for incident adverse health outcomes [34]. Another study also showed that grip strength/body fat mass appears to be the index best associated with MetS [22]. Our study reaffirmed these findings with a significant, graded inverse association between grip strength/body fat mass and MetS (p for trend = 0.045). Future studies need to further identify grip strength indexes that are more relevant to MetS. Although our results suggest that greater muscle strength is a protective factor for MetS, there was no significant difference in overall trend. Muscle strength declines more rapidly during aging than the associated decline in muscle mass [35]. Additionally, the muscle strength and mass of elderly men decreases gradually before the age of 75, and then shows a sharp decline after 75 years old [10]. With an average age 71.3 ± 5.9 years, our population falls in this transition period of sharp decline in muscle strength, which may partly explain our observed nonlinear association between muscle strength and the risk of MetS.

As one of the components of muscle function, physical performance involves the interaction among nerves, balance, and muscle groups. Previous investigations of the relationship between MetS and a variety of physical performance measurements have focused on the general population of older adults, with mixed results. While studies have found that slower walking speed and poor dynamic balance are risk factors for MetS [36, 37], others have not found this association [14, 38]. Sample size issues and the selection of special population (e.g. high-risk population of mobility disability) may have contributed to these differences in results. In our study, there was no significant association between physical performance assessed by walking speed and TUGT and the risk of MetS. Our participants had good functional ability, which may underestimate the risk of MetS in this population. Physical performance is affected by many factors, such as the decline of muscle mass and muscle strength [39], such that it may be the result of MetS rather than the cause. As a multi-system coordination unit, a multitude of components involved in physical performance need to be impaired before it translates to poor overall physical performance. Therefore, redundancy may exist [40].

There is no significant difference between sarcopenia and metabolic syndrome, which may be due to the lack of significant association between the indicators in the current diagnostic criteria of sarcopenia and metabolic syndrome. ASM/ht^2^ provided significant associations with physical disability or frailty, however, because this index is positively correlated with BMI, it has the limitation that subjects with a greater BMI due to a larger amount of fat are less likely to be classified as having sarcopenia [41]. Therefore, its confounding effect may obscure the association with metabolic syndrome.

The significant association between muscle mass and MetS may be explained by a few reasons. First, the plasma glucose uptake stimulated by insulin mainly occurs in skeletal muscle, which highlights its importance in blood glucose control [42]. Second, if the ability of lipid oxidation of skeletal muscle is impaired, it will cause ectopic fat accumulation and may reduce insulin action by interference with insulin signaling [43]. Third, myokine secreted by skeletal muscle fibers, such as IL-6 [44] and irisin [45], has a strong biological effect on reversing metabolic disorder, and the decrease of skeletal muscle mass also causes the decrease of endocrine function.

This study had a few limitations. All participants in the present study were relatively healthy as we did not include participants who were unable to participate in the free annual national physical examination (e.g. those bedridden or with serious disease). Due to this, our results might in fact underestimate of the prevalence of MetS and its associated health impact. Additionally, BIA is not a gold standard for measuring body composition, and its measurement results may not be as accurate as DXA.

In conclusion, our study found that there was a significant, grade inverse association between muscle mass and MetS in a community-dwelling elderly Chinese population. Although higher muscle strength was associated with a lower risk of MetS, it was not linear, and physical performance was not associated with MetS. These results suggest the use of muscle mass improvement training to prevent MetS. The underlying mechanism behind this relationship is still unclear, and future research needs to determine its mechanisms and its ability to predict MetS.

## Supplementary Information

**Additional file 1 **: **Supplementary Table 1.** Logistic regression analyses of the association of grip and ALM quartiles with MetS. **Supplementary Table 2.** Logistic regression analyses of the association of SMI quartiles with MetS in obese and non- obese population.

## Data Availability

The processed data required to reproduce these findings cannot be shared at this time as the data also forms part of an ongoing study, however, it can be obtained from the corresponding author of this article on reasonable request.
