# Supplementary material for: Muscle mass rather than muscle strength or physical performance is associated with metabolic syndrome in community-dwelling older Chinese adults
Source: BMC Geriatr. 2021 Mar 19;21:191. doi: 10.1186/s12877-021-02143-8 (PMC7980667; doi:10.1186/s12877-021-02143-8)
Supplement: Supplementary file 1 — Additional file 1 : Supplementary Table 1. Logistic regression analyses of the association of grip and ALM quartiles with MetS. Supplementary Table 2. Logistic regression analyses of the association of SMI quartiles with MetS in obese and non- obese population. [file 12877_2021_2143_MOESM1_ESM.docx]

Original Articles

**Muscle mass rather than muscle strength or physical performance is associated with metabolic syndrome in community-dwelling older Chinese adults**

Peiyu Song^1, 2^, Peipei Han^1, *^, Yinjiao Zhao^3, *^, Yuanyuan Zhang^2^, Liyan Wang^2^, Zhuoying Tao^1^, Zhengxing Jiang^1^, Shijing Shen^1^, Yunxiao Wu^1^, Jiajie Wu^1^, Xiaoyu Chen^2^, Xing Yu^1^, Yong Zhao^1^, Qi Guo^1^

**Affiliations:**

^1^ Department of Rehabilitation Medicine, Shanghai University of Medicine and Health Sciences, Shanghai, China.

^2^ Department of Rehabilitation Medicine, Tianjin Medical University, Tianjin, China

^3^ Tianjin Key Laboratory of Metabolic Diseases, Key Laboratory of Immune Microenvironment and Disease-Ministry of Education, Department of Physiology and Pathophysiology, Tianjin Medical University, Tianjin, China.

^*^ Peipei Han and Yinjiao Zhao contributed equally to this work and should be considered co-first author.

**Corresponding author:**

Qi Guo, M.D., Ph.D.

Phone: 86-22-8333-6977

FAX: 86-22-8333-6977

E-mail: guoqijp@gmail.com

Address: Department of Rehabilitation Medicine, Shanghai University of Medicine and Health Sciences Affiliated Zhoupu Hospital, 1500 Zhouyuan Road, Pudong New District, Shanghai, 201318, China.

| Supplementary table 1 Logistic regression analyses of the association of grip and ALM quartiles with MetS. | | | | | |
| --- | --- | --- | --- | --- | --- |
|  | Q1 | Q2 | Q3 | Q4 | P for trend |
| Grip(kg) |  |  |  |  |  |
| N | 353 | 353 | 354 | 353 |  |
| Crude | Ref | 0.85 (0.63-1.15) | 0.73 (0.54-0.98) | 0.50 (0.37-0.68) | <0.001 |
| Adjusted model | Ref | 0.95 (0.67-1.34) | 1.00 (0.68-1.45) | 1.24 (0.78-1.98) | 0.160 |
| ALM(kg) |  |  |  |  |  |
| N | 350 | 354 | 354 | 355 |  |
| Crude | Ref | 1.91 (1.41-2.59) | 0.68 (0.50-0.92) | 1.05 (0.77-1.42) | 0.709 |
| Adjusted model | Ref | 1.27 (0.88-1.82） | 0.83 (0.52-1.33) | 1.54 (0.84-2.82) | 0.472 |
| Data are presented as adjusted OR, with 95% CI in parentheses, unless otherwise stated.  Adjusted model is adjusted with age, sex, BMI, smoking status, drinking, occupation, MNA, educational level, family income and physical activity level, CHD, stroke.  Notes: ALM, appendicular lean mass; MNA, Mini-Nutritional Assessment; MetS, metabolic syndrome. | | | | | |

| Supplementary table 2 Logistic regression analyses of the association of SMI quartiles with MetS in obese and non- obese population. | | | | | |
| --- | --- | --- | --- | --- | --- |
|  | Q1 | Q2 | Q3 | Q4 | P for trend |
| Non-obesity |  |  |  |  |  |
| SMI(%) |  |  |  |  |  |
| N | 295 | 295 | 295 | 295 |  |
| Crude | Ref | 0.71 (0.51-0.98) | 0.50 (0.36-0.70) | 0.11 (0.07-0.16) | 0.006 |
| Adjusted model | Ref | 0.68 (0.63-1.41) | 0.37 (0.24-0.59) | 0.08 (0.04-0.14) | 0.009 |
| Obesity |  |  |  |  |  |
| SMI(%) |  |  |  |  |  |
| N | 59 | 58 | 58 | 58 |  |
| Crude | Ref | 4.39 (1.53-12.53) | 1.85 (0.75-4.52) | 1.43 (0.60-3.44) | 0.766 |
| Adjusted model | Ref | 4.34 (1.43-13.18） | 1.62 (0.57-4.58) | 1.61 (0.33.-7.83) | 0.818 |
| Data are presented as adjusted OR, with 95% CI in parentheses, unless otherwise stated.  Adjusted model is adjusted with age, sex, smoking status, drinking, occupation, MNA, educational level, family income and physical activity level, CHD, stroke.  Notes: SMI, skeletal muscle mass index; MNA, Mini-Nutritional Assessment; MetS, metabolic syndrome. | | | | | |
